# Preclinical Assessment of a Metformin–Melatonin Combination: Antinociceptive Synergism

**DOI:** 10.3390/pharmaceutics17081057

**Published:** 2025-08-14

**Authors:** Marcia Yvette Gauthereau-Torres, Jenny Selene Martínez-Guillen, Claudia Cervantes-Durán, Carmen Judith Gutiérrez-García, Daniel Godínez-Hernández, Asdrúbal Aguilera Méndez, Luis Fernando Ortega-Varela

**Affiliations:** 1 División de Estudios de Posgrado, Facultad de Ciencias Médicas y Biológicas “Dr. Ignacio Chávez”, Universidad Michoacana de San Nicolás de Hidalgo, Morelia 58020, Mexico; marcia.gauthereau@umich.mx (M.Y.G.-T.); 0103106j@umich.mx (J.S.M.-G.); 2 Escuela Nacional de Estudios Superiores, Unidad Morelia, Universidad Nacional Autónoma de México (UNAM), Morelia 58000, Mexico; ccervantes@enesmorelia.unam.mx; 3 Departamento de Estudios de Posgrado e Investigación, Tecnológico Nacional de México/Instituto Tecnológico de Morelia, Morelia 58190, Mexico; carmen.gg@morelia.tecnm.mx; 4 Instituto de Investigaciones Químico-Biológicas, Universidad Michoacana de San Nicolás de Hidalgo, Morelia 58030, Mexico; daniel.godinez@umich.mx (D.G.-H.); amendez@umich.mx (A.A.M.); 5 Facultad de Salud Pública y Enfermería, Universidad Michoacana de San Nicolás de Hidalgo, Morelia 58020, Mexico

**Keywords:** analgesic combinations, inflammatory pain, AMPK activation, MT2 receptors, opioidergic pathways

## Abstract

**Background/Objectives:** Pain is a growing public health concern worldwide, and the use of combinations of drugs can improve their analgesic effects while minimizing their adverse effects. Drugs such as metformin (antidiabetic) and melatonin (sleep regulator) have analgesic potential in combination. In this study, we evaluated the pharmacological interaction between metformin and melatonin when orally administered in a rat model, using the formalin test. **Methods:** Female Wistar rats (220–350 g) were injected with 50 µL of 1% formalin in the dorsal surface of the right hind paw. Formalin produces pain-related flinching behavior, and antinociception was evaluated as the reduction in this response. The percentage of the antinociceptive effect was determined after the oral administration of metformin (30–1000 mg/kg), melatonin (10–150 mg/kg), and their combination (MMC). To establish the nature of the interaction, isobolographic analysis was performed in a fixed-dose ratio (0.5:0.5), based on the effective dose 50 (ED_50_**)** values for each drug: metformin (947.46 ± 242.60 mg/kg) and melatonin (126.86 ± 37.98 mg/kg). To evaluate the mechanism of action, the receptor antagonist for metformin compound C (dorsomorphin) for AMPK inhibition, MT1 and MT2 melatonin receptor antagonists (4-P-PDOT, luzindole), and an opioid antagonist (naloxone) were employed. The rotarod test was used to evaluate the safety profile of the combination. **Results:** The metformin–melatonin combination significantly reduced the number of flinches in the second phase of the formalin test. The theoretical ED_50_ for the combination (ED_50_ T) was 537.15 ± 122.76 mg/kg. Experimentally, the ED_50_ (ED_50_ E) was significantly lower (360.83 ± 23.36 mg/kg), indicating a synergistic interaction for the combination involving opioidergic pathways, MT2 receptors, and AMPK activation. **Conclusions:** Oral metformin–melatonin coadministration could provide a therapeutic alternative for inflammatory pain.

## 1. Introduction

Pain is a significant public health concern worldwide, which has led to an elevated demand for pharmacological strategies to manage this condition. It has been estimated that almost 20% of adults suffer from pain [1]. Combinations of drugs are widely used in order to improve their analgesic treatments and decrease their adverse effects, thus achieving better long-term pain control [2].

Pleiotropic effects are drug actions that differ from those for which the agent was specifically designed, which may be variably related to the primary mechanism of action of the medication [3]. Drugs with pleiotropic effects such as metformin (antidiabetic) and melatonin (sleep regulator) are promising candidates for combination therapies, as both have demonstrated analgesic potential [4,5].

Metformin is a safe and well-tolerated oral biguanide, broadly employed for treatment of type 2 diabetes [6]. In addition to its glucose lowering effects, metformin contributes to weight loss in overweight individuals [7] and to reductions in inflammation and plasma lipid levels [8]. A recent systematic review of preclinical and clinical studies showed that metformin has structural protective, anti-inflammatory, and analgesic effects in osteoarthritis [6]. These pleiotropic effects of metformin are mainly driven by the activation of AMP-activated protein kinase (AMPK), which in turn diminishes mTORC1 signaling in nociceptors [9,10].

Another drug with pleiotropic effects is melatonin (N-acetyl-5-methoxytryptamine), an endogenous neurohormone synthesized and secreted mainly by the pineal gland [11]. Traditionally, melatonin is known for its neurobiological role in regulating the sleep–wake cycle [12]. However, melatonin also exhibits antioxidant and anti-inflammatory properties, acting as a free radical scavenger during inflammation and injury [13,14]. In addition, it has been demonstrated that melatonin exerts antinociceptive effects by acting at both the spinal cord and supraspinal levels through the activation of MT1 and MT2 receptors [15]. Moreover, melatonin indirectly activates the opioid system and modulates GABA(A) receptor function, contributing to pain modulation [16]. Therefore, the aim of this study was to evaluate the possible antinociceptive interactions between metformin and melatonin in an inflammatory pain model—specifically, the rat formalin test—as well as to explore the underlying potential mechanism of their combined administration.

## 2. Materials and Methods

### 2.1. Animals

Female Wistar rats (220–350 g) were used to perform the experiments, since no differences have been observed between males and females in previous studies with this rat strain or formalin concentration [17,18]. The animals were housed under controlled conditions of temperature (22 ± 2 °C) and light (12 h: 12 h) and had access to food and water ad libitum. All experiments were carried out according to the guidelines on ethical standards for investigation on experimental pain in animals [19] and Mexican regulations [20]. The Instituto de Investigaciones Químico Biológicas Bioethics Committee approved all experiments (Code: IIQB-05/2023). The minimum necessary dose for the intensity of noxious stimuli and number of animals were both used, in order to determine consistent effects of the drug treatment [21]. The rats were euthanized in a CO_2_ chamber when the experiments were completed [22].

### 2.2. Drugs

Metformin, melatonin, compound C (dorsomorphin, AMPK-selective inhibitor), 4P-PDOT (4-phenyl-2-propionamidotetralin, MT2 melatonin receptor-specific antagonist), luzindole (non-selective MT1/MT2 receptor antagonist), naloxone (opioid antagonist), and dimethyl sulfoxide were purchased from Sigma (St. Louis, MO, USA). Carboxymethylcellulose and formaldehyde were purchased from Meyer (Mexico City, Mexico). Metformin and melatonin were dissolved in 1% carboxymethylcellulose. Compound C, 4P-PDOT, luzindole, and naloxone were dissolved in dimethyl sulfoxide.

### 2.3. Formalin-Induced Acute Nociception

As described by Dubuisson and Dennis (1977) [23], acute nociception was assessed using the formalin test, with some modifications [24,25]. The rats were placed for 30 min in open clear acrylic cylinders to allow them to acclimatize to their surroundings; then, they received the formalin injection. Rats were injected in the dorsum of the right hind paw with 50 µL of diluted formalin (1% or 0.5%) or vehicle (carboxymethylcellulose or dimethyl sulfoxide) with a 30-gauge needle, while they were gently restrained. After formalin injection, nociceptive behavior was observed immediately. Each cylinder had mirrors to enable unhindered observation. Nociceptive behavior was quantified as the number of flinches of the injected paw/hindquarters during 1 min periods every 5 min, up to 60 min after injection. Flinching was characterized by a rapid and brief withdrawal or as flexing of the injected paw/hindquarters. Formalin-induced flinching behavior was biphasic [24,25], with an initial acute phase (0–10 min), followed by a relatively short quiescent period and then a prolonged tonic response (15–60 min).

### 2.4. Study Design

In the present study, animals were orally administered with increasing concentrations of either metformin (30, 100, 300, and 1000 mg/kg), melatonin (10, 30, 75, and 150 mg/kg), the MMC (Table 1), or a unique dose of vehicle 60 min before subcutaneous formalin injection (50 μL). In order to determine the possible mechanism of action of MMC on antinociception, compound C (dorsomorphin, AMPK-selective inhibitor, 3 mg/kg), 4P-PDOT (4-phenyl-2- propionamidotetralin, MT2 melatonin receptor-specific antagonist, 1 mg/kg), luzindole (non-selective MT1/MT2 receptor antagonist, 1 mg/kg), and naloxone (opioid antagonist, 1 mg/kg) were used. The inhibitor and antagonists (50 µL) were administered 10 min before MMC ED_50_ and, after 60 min, rats received a subcutaneous formalin injection. The inhibitor and antagonist doses were chosen based on previous studies [26,27].

### 2.5. Data Analysis

The results are presented as the mean ± S.E.M. (standard error of the mean) of six animals per group (a total of 22 groups). The time courses of the antinociceptive responses of metformin and melatonin separately and in combination were constructed by plotting the mean number of flinches as a function of time. To compare three or more experimental groups, we used two-way repeated measures analysis of variance (ANOVA), followed by a Student–Newman–Keuls test. The area under the curve (AUC) of the number of flinches against time, for each case, was calculated using the trapezoidal method. Dose–response data are presented as the percent of antinociception, calculated from the AUC of phase 2 of the formalin test, according to the following equation [28]:

% Antinociception = AUC vehicle − AUC drugs × 100,

AUC vehicle.

The dose–response curves were constructed, and the experimental data points were fitted using least-squares linear regression. The ED_50_ ± standard error (S.E.M.) was calculated according to the method described by Tallarida (2001) [29]. It has been previously demonstrated that isobolographic analysis is a useful tool for evaluation of the interaction between analgesic drugs [30]. In the present study, this method was employed to determine the nature of drug interactions between metformin and melatonin when administered orally. In this analysis, we assumed that the combination of drugs would represent equally effective doses of the individual drugs. Thus, from the dose–response curves of each individual agent, the dose resulting in 50% of the effect (ED_50_ value) can be determined.

To determine whether the interaction between both drugs given in combination was additive, synergistic, or antagonistic, the theoretical additive ED_50_ value was estimated from the log dose–response curve of each compound administered individually, assuming that the observed combined effect results from the summation of the individual effects of each component. This theoretical ED_50_ value of the combination was then compared with the experimental ED_50_ values to determine whether there was a significant difference [28,30]. The experimental ED_50_ value was obtained via oral coadministration of fractions of the ED_50_ respective value for each drug: 1/2, 1/4, 1/8, and 1/16. Isobolographic analysis was then used to characterize the antinociceptive interaction between metformin and melatonin in the formalin test. The theoretical and experimental ED_50_ values of the studied combinations were also compared by calculating the interaction index (γ), defined as follows [31]:

γ = ED value of combination (experimental),

ED value of the combination (theoretical).

The interaction index indicates the proportion of the ED_50_ value of the individual compounds that accounts for the corresponding ED_50_ value in the combination; that is, values near to unity correspond to an additive interaction, values higher than 1 indicate an antagonistic interaction, and values lower than 1 indicate a synergistic interaction [2].

### 2.6. Assessment of Motor Activity

Animals were trained for 2 days to walk on an accelerating rotarod apparatus (Panlab 8500, Cornellá Barcelona, Spain) at 4 rpm. The speed was gradually increased until reaching 40 rpm in 10 min [32]. The trained animals received an oral dose of vehicle (carboxymethylcellulose 1%), MMC ED_50_ (473.72 mg/kg of metformin and 63.42 mg/kg of melatonin), or diazepam (10 mg/kg) 60 min before testing. Diazepam (10 mg/kg, p. o.) was used as a positive control, as it produces motor side effects [33]. Motor performance was evaluated as the latency until fall from the rotarod apparatus, determined from the mean time in three trials for each rat at each time. The results of these tests were followed by one-way ANOVA followed by the Student–Newman–Keuls post hoc test. Graphs were constructed using GraphPad Prism 8.0.

## 3. Results

### 3.1. Antinociceptive Effect of Metformin and Melatonin

**Figure 1:**
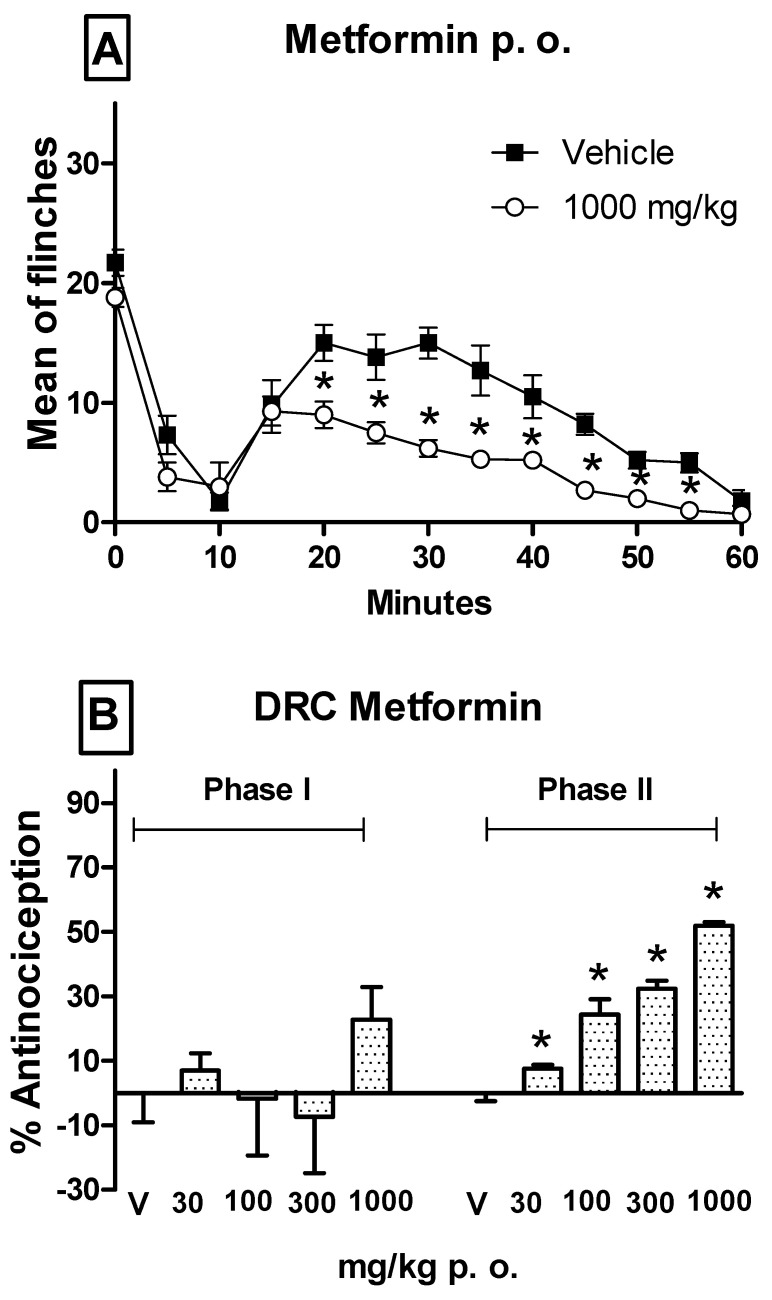
(**A**) Temporal course of the formalin (1%) test obtained after oral administration (p. o.) of metformin (1000 mg/kg −1 h). Data are expressed as the mean number of paw flinches ± S.E.M. of six animals. Significant treatment differences were observed only in phase 2 (11–60 min) vs. the vehicle group, * *p* < 0.05, according to the two-way repeated measures ANOVA followed by a Student–Newman–Keuls test. (**B**) Dose–response curves (DCR) in the second phase of formalin test for metformin (30, 100, 300, and 1000 mg/kg). Data are expressed as the percentage of antinociception. Bars represent the mean ± S.E.M. of six animals. Significantly different values were found from the vehicle group, * *p* < 0.05, using a one-way ANOVA followed by a Student–Newman–Keuls test.

**Figure 2:**
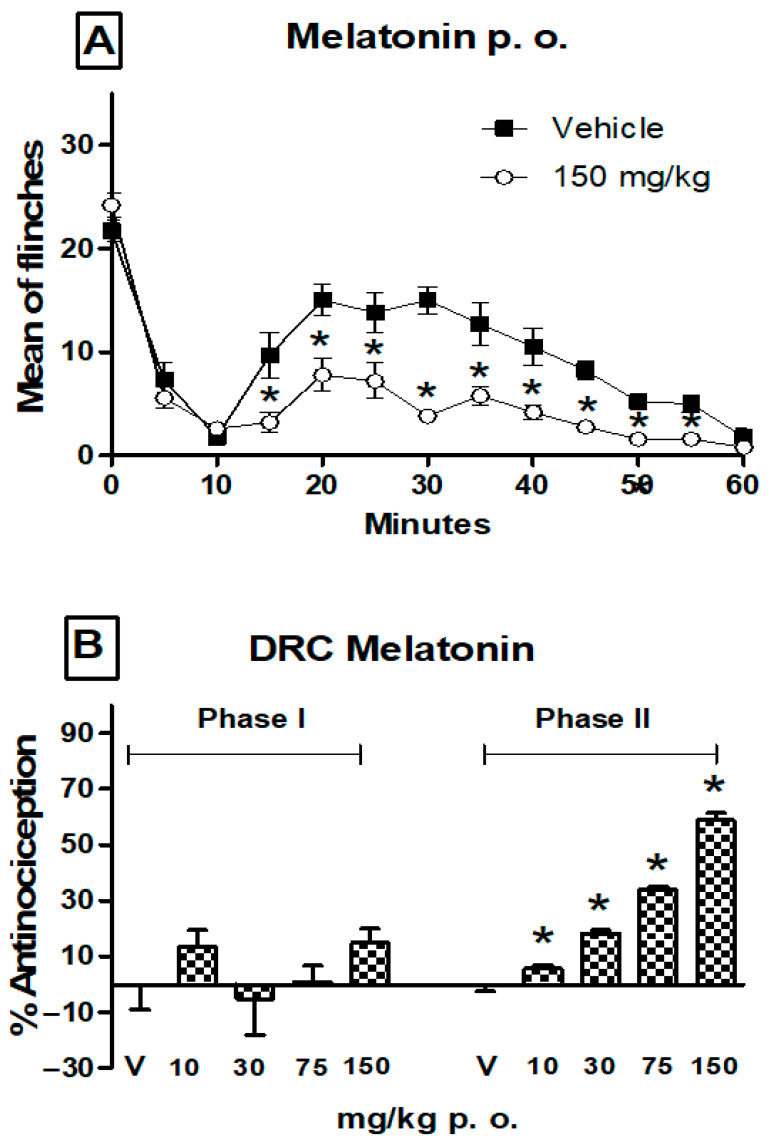
(**A**) Temporal course of the formalin (1%) test obtained after oral administration (p. o.) of melatonin (150 mg/kg −1 h). Data are expressed as the mean number of paw flinches ± S.E.M. of six animals. Significant treatment differences were observed only in phase 2 (11–60 min) vs. the vehicle group, * *p* < 0.05, according to the two-way repeated measures ANOVA followed by a Student–Newman–Keuls test. (**B**) Dose–response curves (DCR) in the second phase of the formalin test for melatonin (10, 30, 75 and 150 mg/kg). Data are expressed as the percentage of antinociception. Bars represent the mean ± S.E.M. of six animals. Significantly different values were found from the vehicle group, * *p* < 0.05, according to a one-way ANOVA followed by a Student–Newman–Keuls test.

Figure 1 and Figure 2 show the typical time course of the formalin test and the effects of orally administered metformin and melatonin given separately. Individual administration of either metformin or melatonin significantly decreased the number of flinching episodes during both phases 1 and 2, when compared with vehicle-treated rats in the formalin test. However, as neither drug produced a dose-dependent reduction in nociceptive flinching behavior in phase 1, only phase 2 was submitted to additional analysis. The maximum percentage of antinociception observed in phase 1 is shown in Table 2. In the dose–response curve for metformin, doses of 30–1000 mg/kg were orally administered. In phase 2, a maximum antinociceptive effect of approximately 52% was observed, and the response was dose-dependent (Figure 1). For melatonin, doses of 10–150 mg/kg were administered orally (Figure 2). The antinociceptive effect of melatonin in Phase 2 was also dose-dependent, with a maximum efficacy of approximately 60%.

### 3.2. Antinociceptive Effect of Metformin–Melatonin Combination

**Figure 3:**
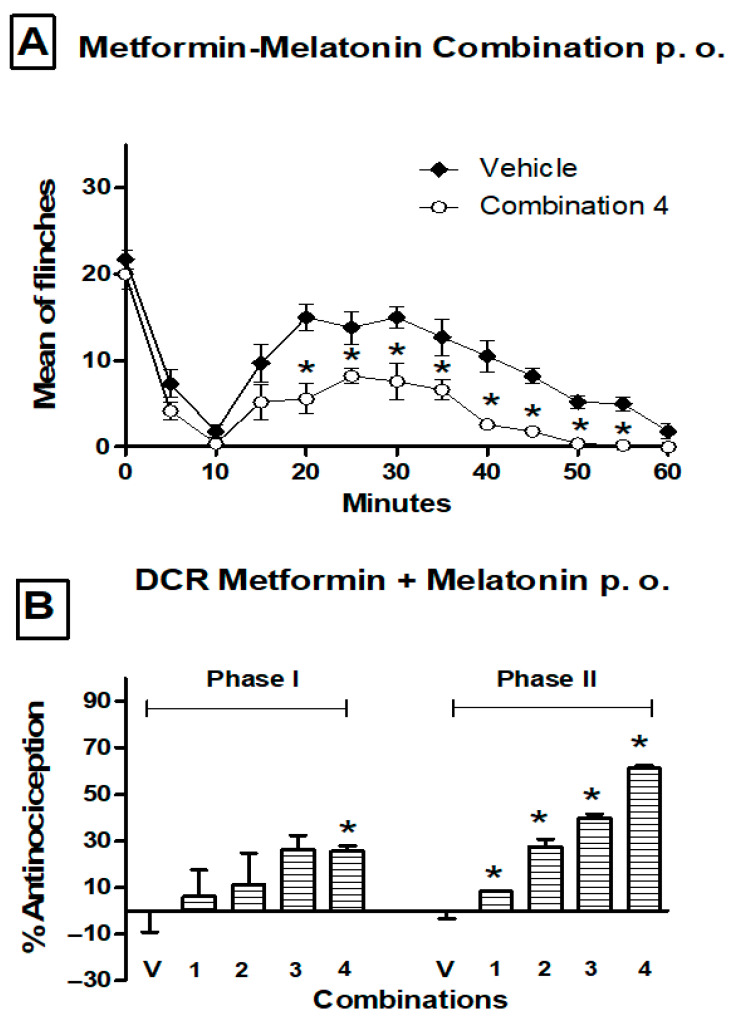
(**A**) Temporal course of the formalin (1%) test obtained after oral administration (p. o.) of a combination of 473.72 mg/kg of metformin and 63.42 mg/kg of melatonin (−1 h). Data are expressed as the mean number of paw flinches ± S.E.M. of six animals. Significant treatment differences were observed only in phase 2 (11–60 min) vs. the vehicle group, * *p* < 0.05, according to the two-way repeated measures ANOVA followed by a Student–Newman–Keuls test. (**B**) Dose–response curves (DRC) in the second phase of the formalin test for combination of MMC, according to the doses in Table 1. Data are expressed as the percentage of antinociception. Bars represent the mean ± S.E.M. of six animals. Significantly different values were found from the vehicle group, * *p* < 0.05, according to the one-way ANOVA followed by a Student–Newman–Keuls test.

**Figure 4:**
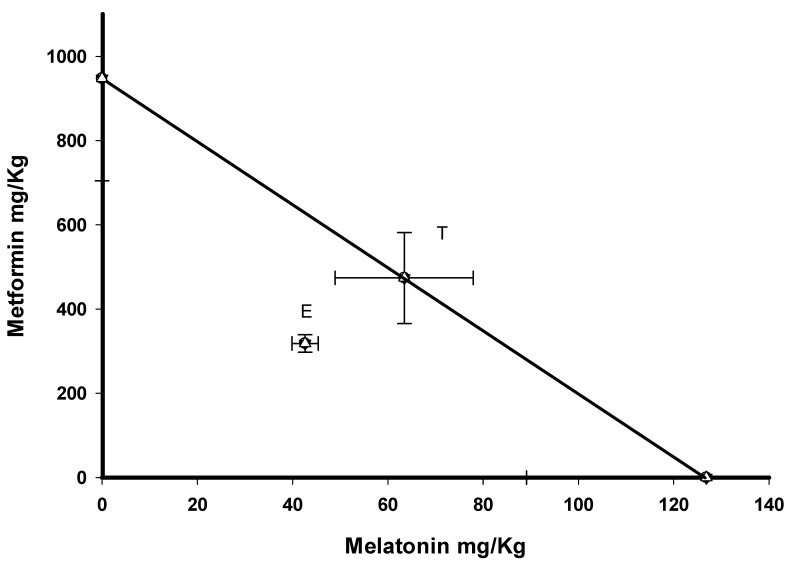
The isobologram of the oral (p. o.) interaction of metformin and melatonin is shown. Horizontal and vertical bars indicate the S.E.M. The oblique line between the x- and y axes is the theoretical additive line. The point in the middle of this line, indicated by T, is the theoretical additive point calculated from the individual drug ED_50_ values. The experimental point, indicated by E, is the ED_50_ actually observed with the combination. The experimental ED_50_ point lies far below the additive line, indicating a significant synergistic interaction (*p* < 0.05), as determined by the Student’s *t*-test.

After oral intake, co-administration of metformin and melatonin induced a dose-dependent increase in the percentage of antinociception in phase 2 (Figure 3). After computing the ED_50_ values from the dose–response curve, these were submitted to isobolographic analysis, showing that the experimental values were lower than those expected from a purely additive interaction (Figure 4). The theoretically additive dose line depicts all points of metformin and melatonin dose combinations yielding an effect of 50% according to an additive interaction. The experimental ED_50_ values of the metformin and melatonin combination were located clearly below the theoretically additive dose line, indicating a synergistic interaction between metformin and melatonin after oral administration (Figure 4).

Comparison of the experimental and theoretical ED_50_ values using the Student’s *t*-test yielded statistically significant differences (*p* < 0.05) (Table 3). For this combination, the interaction index (γ) was lower than 1; analysis of the interaction index (Table 3), showed an increase in potency after oral administration—almost 1.3-fold for the combination employed (γ = 0.67)—highlighting their synergistic effects.

### 3.3. Action Mechanisms of the Combination

When the rats were pretreated with 4-P-PDOT (1 mg/Kg s.c.), this selective antagonist of the MT2 melatonin receptor caused a significant reduction in the metformin–melatonin combination ED_50_ effect in the second phase of the formalin test (*p* ≤ 0.05). In contrast, luzindole pretreatment (non-selective MT1/MT2 receptor antagonist −1 mg/Kg s.c.) did not significantly affect the antinociceptive effect of the combination. 

**Figure 5:**
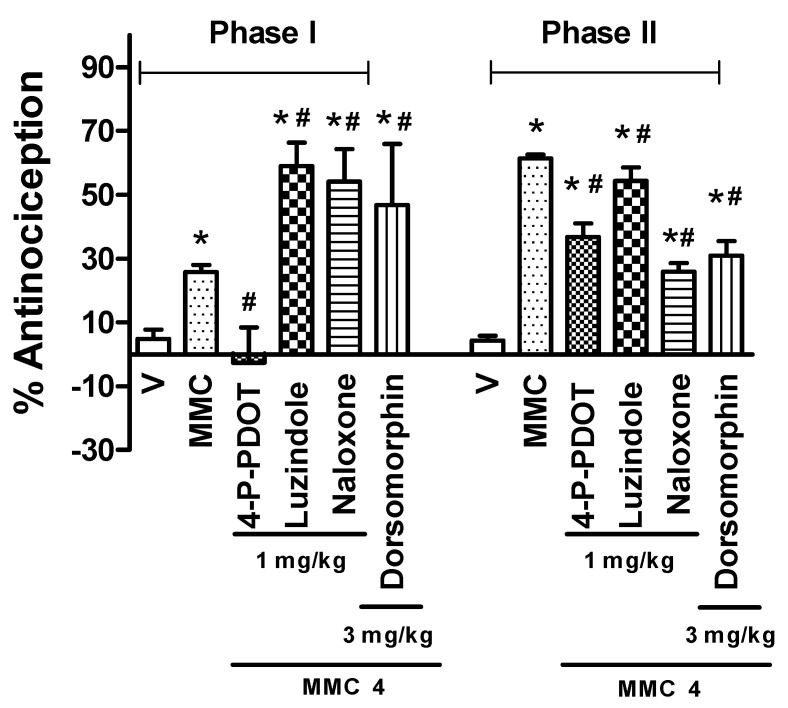
Effects of the administration (s. c.) of selective MT2 receptor antagonist (4-P-PDOT) and non-selective (luzindole) MT1-MT2 melatonin receptor antagonist, opioid receptor antagonist (naloxone), and AMPK blocker (dorsomorphin) (−70 min) and the combination of 473.72 mg/kg of metformin and 63.42 mg/kg of melatonin (MMC) (−1 h) in rats, during phase 2 of the formalin test. Data are expressed as the percentage of antinociception of the maximum possible effect. Bars represent the mean ± S.E.M. of six animals. * Significantly different (*p* < 0.05) from the vehicle group (V) and # significantly different (*p* < 0.05) from the MMC, according to a one-way ANOVA followed by a Student–Newman–Keuls test.

In addition, pretreatment with the opioid receptor antagonist naloxone (1 mg/Kg s.c.) decreased the percentage of antinociception of the combination in the formalin inflammatory phase (phase 2). In the same sense, in the group pretreated with the AMPK inhibitor dorsomorphin (3 mg/Kg s.c.), the antinociceptive effect of the metformin–melatonin combination was effectively blocked (Figure 5).

**Figure 6:**
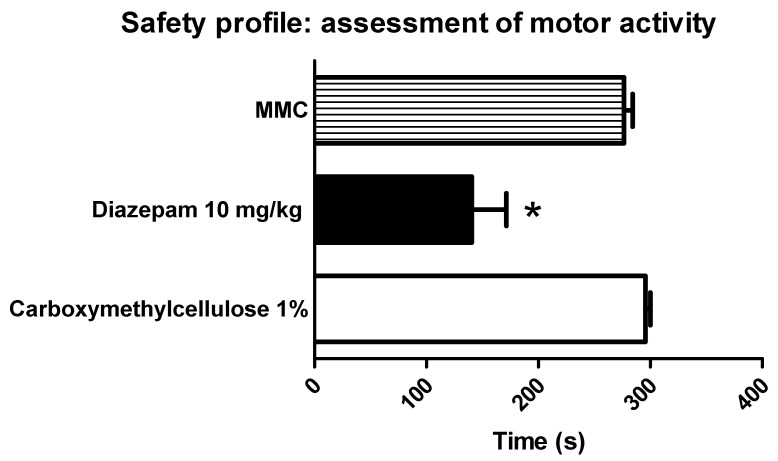
Safety profile of the combination of 473.72 mg/kg of metformin and 63.42 mg/kg of melatonin (MMC) in the rotarod test. Data are expressed as the time in seconds of rats placed on the rotarod for 5 min at a speed of 40 revolutions per minute (rpm). Bars represent the mean ± S.E.M. of six animals. * Significantly different (*p* < 0.05) from the carboxymethylcellulose 1% (vehicle group), according to a one-way ANOVA followed by a Student–Newman–Keuls test.

Additionally, the rotarod performance of the ED_50_ of the metformin–melatonin combination was no different from the control group (carboxymethylcellulose 1%), but it was significantly different from the diazepam treatment group (10 mg/Kg) (Figure 6).

## 4. Discussion

### 4.1. Neurogenic Phase of the Test

In the current study, we first examined the absence of dose-dependent effects after administration of metformin and melatonin, either individually or in combination, in the first phase of the formalin test. This suggests that, at this initial stage of the nociceptive model, the pharmacological effects of both substances do not exhibit a linear relationship with the administered dose, which could suggest that the effect of the drugs does not involve the blockade of initial neurogenic activation in the formalin test, and that metformin and/or melatonin could be acting on peripheral inflammation and central sensitization, which are responsible for the pain in the second phase of the formalin test [34,35].

### 4.2. Antinociceptive Effect of Metformin

We observed an antinociceptive effect of the oral administration of metformin in rats, which was more clearly manifested in the second phase of the formalin test. Metformin is a first-line hypoglycemic agent in the treatment of type 2 diabetes mellitus; it also has additional benefits such as cardiovascular protection and potential anti-aging effects [36]. Metformin is known to be an activator of the AMPK enzyme complex—an enzyme that regulates cellular energy metabolism—leading to a reduction in hepatic glucose synthesis, an increase in glucose uptake by skeletal muscle, and enhanced insulin sensitivity. Furthermore, metformin can reduce intestinal glucose absorption and improve the lipid profile in patients with type 2 diabetes without causing significant hypoglycemia when used as a monotherapy [37,38,39].

Regarding nociception, metformin produces analgesic effects by modulating different cellular mechanisms, mainly through the activation of AMP-activated protein kinase (AMPK). This activation in the neurons of the dorsal root ganglia and in the spinal cord inhibits proinflammatory pathways, especially NF κB, decreasing the production of cytokines such as TNF α and IL 6, which reduces central sensitization [40]. Meanwhile, metformin is also capable of increasing the release of anti-inflammatory cytokines (IL-4, IL-10) [8,10,41]. In addition, phosphorylated AMPK promotes the regulation of ion channels involved in pain, such as NaV1.7 and TRPA1, reducing neuronal excitability [42]. In inflammatory pain models, metformin also enhances serotonin release and activates 5 HT_1_A/1B D receptors, which contributes to its antinociceptive effect [43]. Furthermore, it has been observed that it reduces glutamatergic transmission in pathological pain at the spinal level by restoring synaptic plasticity and decreasing markers such as c-Fos, which attenuates the perception of pain [44]. In this sense, our results correlate with the analgesic effects of metformin in humans [45,46], and are also in line with other findings demonstrating that metformin is able to reduce inflammatory nociception and prevent neuropathic pain originating in the hot plate model due to spinal cord injury or streptozotozine-induced diabetic neuropathy in rats [5,46,47].

Comparative studies have been conducted on the analgesic effects of metformin in different types of pain with respect to some non-steroidal analgesics, which have shown that it can have an analgesic potency even higher than aspirin [48,49].

In this study, metformin doses in the range of 30–1000 mg/Kg were used and a maximum effect of 51.8% was observed following administration, which is consistent with previous reports [5]. Metformin did not show high or dose-dependent efficacy in phase 1 of the formalin trial, which could indicate that the drug does not directly affect nociceptor activity but, instead, its main effect could be due to the inhibition of peripheral sensitization, thus decreasing the inflammatory response to injury [8,41,50,51]. In contrast to our results, other studies did not attribute significant analgesic action to metformin, mainly because they did not observe analgesic effects at the doses they used (5–15% of the ones we used) [47,52,53]. In fact, another study also used low doses of metformin and some other hypoglycemic agents that are believed to have the ability to block the analgesic effect of diclofenac (an NSAID) [54]. The different experimental models and conditions used could explain these discrepancies, which clearly indicates the need for further research into the antinociceptive effects of metformin.

### 4.3. Antinociceptive Effect of Melatonin

Orally administered melatonin produced dose-related antinociception in phase 2 of the 1% formalin test in rats. This nociceptive behavior resulted in a decrease in the number of flinches of the right hind leg (i.e., where the formalin was applied). According to some authors, the antinociceptive action of melatonin is due to the fact that it acts through mechanisms mediated by membrane receptors MT1 and MT2 coupled to the Gi/o protein and by nuclear receptors [15,55,56]. By binding to these receptors, adenylyl cyclase is inhibited, which reduces intracellular cAMP levels and prevents the activation of protein kinase A (PKA) and CREB phosphorylation, thus modulating neuronal excitability [15]. At the level of the nervous system, the activation of these receptors in the spinal cord and supraspinal structures (thalamus, hypothalamus, dorsal horn) causes the opening of K^+^ channels and inhibition of Ca^2+^ channels, thus reducing the excitability of nociceptive neurons and central sensitization. Likewise, melatonin indirectly interacts with opioid, GABAergic, serotonergic, adrenergic, and benzodiazepine systems, blocking inflammatory pathways (such as NO-cGMP), decreasing the expression of COX 2 and iNOS, and acting as a potent anti-inflammatory and antioxidant [30,31].

Melatonin plays a significant role in antinociception against inflammatory and pathological pain. Melatonin inhibits adenylate cyclase, decreasing intracellular cAMP, reducing the elevated expression of FN-κB, and decreasing proinflammatory cytokines such as IL-6 y FNT-α. This effect could be due to the decrease in the release of prostaglandins and NO-synthase at the site of injury for inflammatory pain. It also depresses nociceptive discharges from spinal dorsal horn neurons, regulating neuropathic pain [27,57,58,59]. For this drug, the maximum observed oral effect in phase 1 of the formalin trial was 22.7% while, in phase 2, it was 58.2%; this effect has been reported by other authors [27]. All of this supports our findings in the work.

### 4.4. Antinociception of the Metformin–Melatonin Combination

In this study, through measuring the antinociceptive effect quantitatively in the rat formalin test, isobolographic analysis demonstrated a significant synergistic interaction between metformin and melatonin after oral administration. Metformin has been tested in analgesic combinations with fluoxetine [60], curcumin [61,62], ibuprofen, and naproxen [63]. On the other hand, melatonin has also been co-administrated with morphine [64,65], diazepam [64], paymitoylethanolamyde [66,67], paracetamol [66], pregabalin [68], and tramadol [69]. In these cases, both drugs have been shown to have significant analgesic effects, which is consistent with our findings regarding the antinociceptive effects of metformin and melatonin individually, and even more so when combined.

Metformin has previously been found to be highly effective in combination with melatonin, improving glucose metabolism [70,71]; in rat models of polycystic ovary syndrome [72,73]; against neoplasm development, such as skin carcinogenesis [74,75], adrenocortical carcinoma [76], or mammary carcinogenesis [77,78]; and showing radioprotective effects [79,80]. However, to the authors’ knowledge, this is the first report focused on the synergistic antinociceptive interaction between metformin and melatonin.

The interaction index (γ) is a measure of the degree of synergism or sub-additivity that occurs when two drugs are co-administered [2,25]; the γ value for this combination was 0.67, indicating that lower doses of each group were required to produce antinociception. The resulting effect was about 1.3 times higher than that expected for the sum of the effects of the individual components.

Differences in the pharmacokinetics of both drugs may be argument against a sustained interaction between them; melatonin maximum plasma levels are observed at 90 min, while metformin reaches this maximum concentration at 3 h. However, significative efficacy of the metformin–melatonin combination has been reported for at least 180 min in antihyperglycemic assessments [71]. In addition, the experimental effective dose 50 reported in Table 3 of 360.3 mg/Kg (313 + 47 mg/Kg of metformin and melatonin, respectively) might seem elevated; however, according to previous reports, the translation of dosages from animal models to human clinical trials based only in body weight or body area surface can lead to inappropriate dose recommendations [81]. Other validated approaches with physiologic and pharmacokinetic data will be needed to determine the therapeutic ranges of a metformin–melatonin combination in humans [82].

The mechanism of the observed synergism may be attributed to the different sites of action of metformin and melatonin, as well as to the multiple antinociceptive mechanisms of action of both drugs. Our experimental results demonstrated that 4-P-PDOT antagonized the antinociceptive effect of the combination, while luzindole did not eliminate the effect of the coadministration; in this sense, our present results may imply that MT2 receptor-dependent mechanisms are involved. In agreement with this, several studies have shown that MT2 receptor activation produces antinociception in inflammatory pain and tactile allodynia models [4,27,83]. In fact, Lopez-Canul et al. (2015) [84] demonstrated that selective MT2 receptor partial agonists have analgesic properties through modulation of brainstem-descending antinociceptive pathways.

In addition, we investigated the effects induced by the opioid antagonist naloxone on the antinociceptive activity of the metformin–melatonin combination. Pretreatment with naloxone markedly diminished the efficacy of the combination. These results agree with previous observations of the activation of opioidergic mechanisms in the antinociceptive activities of metformin [5] and melatonin [4]. Since neither metformin nor melatonin bind to opioid receptors, an increase in ß-endorphin in the central nervous system is indicated as a potential common mechanism. In this sense, Cheng et al. (2006) [85] reported the oral intake of metformin decreased the plasma glucose in STZ-induced diabetic rats with a parallel increase in plasma beta-endorphin; accordingly, Shavali et al. (2005) [86] observed a time-dependent release of beta-endorphin, an endogenous opioid peptide, by melatonin from mouse pituitary cells in culture.

There is a body of evidence linking AMPK activation with a reduction in nociceptor excitability and preventing the development of tactile allodynia [39], as metformin is recognized as an AMPK activator. In the present study, the pretreatment with dorsomorphin, the selective AMPK inhibitor, yielded a significant decrease in the antinociceptive effect of the metformin–melatonin combination. This finding agrees with previous data reported by Russe et al. (2013) [87], who indicated that AMPK activation exerts antinociceptive and anti-inflammatory effects. Comprehensive data on pain indicate that metformin targets the master switch kinase (AMPK), which in turn decreases the activity of the mTORC1 and MAPK signaling pathways in nociceptors, resulting in a reversal of both acute and chronic pathological pain [10,88]. Several studies have suggested that melatonin may have beneficial metabolic effects due to the activation of AMP-activated protein kinase [89,90]. However, it is unclear whether melatonin directly modulates AMPK signaling in nociceptive pathways.

Competitive inhibition of dorsomorphin with AMPK in the spinal cord disrupts the negative feedback that reduces opioid analgesia signaling; in turn, melatonin primarily activates MT receptors and promotes AMPK activation in various tissues including the spinal cord, supporting mitochondrial function and limiting inflammatory responses. In addition, it attenuates the activation of the NLRP3 inflammasome in spinal microglia, reducing the release of IL-1β linked to morphine tolerance. The interaction between dorsomorphin and melatonin suggests a dual modulator of AMPK in pain: while dorsomorphin inhibits AMPK and can worsen inflammation, melatonin reactivates or maintains its function, counteracting tolerance and neural damage. This points to the importance of a balance between AMPK inhibition and activation to optimize analgesia and minimize adverse effects in pain management [91].

Formalin injection produces acute (short-lasting) and chronic (long-lasting) nociception, which manifests as secondary allodynia and hyperalgesia [4]. In studies conducted in long-term pain models, it has been observed that the administration of antinociceptive drugs prior to the administration of 0.5% or 1% formalin prevents the development of long-term nociceptive behaviors such as allodynia and hyperalgesia [27]. In this sense, a similar behavior could be expected in the case of the combination of metformin and melatonin.

The higher dose of the metformin–melatonin combination (473.72 + 63.42 mg/Kg, respectively) did not alter the motor performance in the rotarod test (Figure 6). Higher doses of metformin and melatonin with no motor impairing actions have been reported elsewhere [4,5,27]. Furthermore, no side effects were observed during the present experiments, suggesting that this combination has a favorable side-effect profile. In contrast, our results also agree with previous observations on the motor-impairing side effects of diazepam [33,92].

## 5. Conclusions

The present findings demonstrated that the metformin–melatonin combination produces functional synergic activity in the rat formalin test to reduce inflammatory pain. These antinociceptive effects may be mediated by the participation of MT2 receptors, opioidergic pathways, and AMPK activation. The reduction in almost a third of the dose requirements with no exacerbation of side effects suggests that this combination could be useful against inflammatory pain states.

## Figures and Tables

**Table 1:** Experimental doses for combination p. o.

Metformin mg/kg		Melatonin mg/kg
1.	59.21	7.92
2.	118.43	15.85
3.	236.86	31.71
4.	473.72	63.42

**Table 2:** Maximum effect of oral administration of metformin and melatonin, alone or in combination, in phase 1 in the formalin test.

Treatment	% Antinociception Maximum Effect in Phase 1, Oral Route
Metformin 1000 mg/kg	22.7 ± 10.2
Melatonin 150 mg/kg	0.4 ± 7.3
Metformin (473.72 mg/Kg) and melatonin (63.42 mg/Kg)	24.5 ± 2.3

**Table 3:** Parameters of isobolographic analysis for oral metformin and melatonin.

Drugs Administered Orally				
DE50 ± S.E.M. Metformin (mg/Kg)	DE50 ± S.E.M. Melatonin (mg/Kg)	DE50 ± S.E.M. Theoretical (mg/Kg)	DE50 ± S.E.M. Experimental (mg/Kg)	Interaction Index (γ) ± S.E.M.
947.46 ± 242.58	126.85 ± 37.97	537.15 ± 122.76	360.83 ± 23.36	0.672 ± 0.1596

S.E.M. = standard error of the mean ED50 = effective dose 50.

## Data Availability

The original contributions presented in this study are included in this article; further inquiries can be directed to the corresponding author.

## References

[1] Goldberg D.S., McGee S.J. (2011). Pain as a Global Public Health Priority. BMC Public Health.

[2] Tallarida R.J. (2002). The Interaction Index: A Measure of Drug Synergism. Pain.

[3] Sica D.A. (2011). Are There Pleiotropic Effects of Antihypertensive Medications or Is It All About the Blood Pressure in the Patient With Diabetes and Hypertension?. J. Clin. Hypertens..

[4] Ambriz-Tututi M., Granados-Soto V. (2007). Oral and Spinal Melatonin Reduces Tactile Allodynia in Rats via Activation of MT2 and Opioid Receptors. Pain.

[5] Augusto P.S.A., Braga A.V., Rodrigues F.F., Morais M.I., Dutra M.M.G.B., Batista C.R.A., Melo I.S.F., Costa S.O.A.M., Goulart F.A., Coelho M.M. (2019). Metformin Antinociceptive Effect in Models of Nociceptive and Neuropathic Pain Is Partially Mediated by Activation of Opioidergic Mechanisms. Eur. J. Pharmacol..

[6] Lim Y.Z., Wang Y., Urquhart D.M., Estee M.M., Wluka A.E., Heritier S., Cicuttini F.M. (2023). Metformin for Knee Osteoarthritis with Obesity: Study Protocol for a Randomised, Double-Blind, Placebo-Controlled Trial. BMJ Open.

[7] Malin S.K., Kashyap S.R. (2014). Effects of Metformin on Weight Loss: Potential Mechanisms. Curr. Opin. Endocrinol. Diabetes Obes..

[8] Saisho Y. (2015). Metformin and Inflammation: Its Potential Beyond Glucose-Lowering Effect. Endocr. Metab. Immune Disord.-Drug Targets.

[9] Hasanvand A. (2022). The Role of AMPK-Dependent Pathways in Cellular and Molecular Mechanisms of Metformin: A New Perspective for Treatment and Prevention of Diseases. Inflammopharmacology.

[10] Baeza-Flores G.D.C., Guzmán-Priego C.G., Parra-Flores L.I., Murbartián J., Torres-López J.E., Granados-Soto V. (2020). Metformin: A Prospective Alternative for the Treatment of Chronic Pain. Front. Pharmacol..

[11] Mahmood D. (2018). Pleiotropic Effects of Melatonin. Drug Res..

[12] Brzȩczek M., Słonka K., Hyla-Klekot L. (2016). Melatonina-Hormon o Plejotropowym Działaniu. Pediatr. I Med. Rodz..

[13] Dominguez-Rodriguez A., Abreu-Gonzalez P., Reiter R.J. (2012). Melatonin and Cardioprotection in the Acute Myocardial Infarction: A Promising Cardioprotective Agent. Int. J. Cardiol..

[14] Nabavi S.M., Nabavi S.F., Sureda A., Xiao J., Dehpour A.R., Shirooie S., Silva A.S., Baldi A., Khan H., Daglia M. (2019). Anti-Inflammatory Effects of Melatonin: A Mechanistic Review. Crit. Rev. Food Sci. Nutr..

[15] Srinivasan V., Lauterbach E.C., Yu Ho K., Acuna-Castroviejo D., Zakaria R., Brzezinski A. (2012). Melatonin in Antinociception: Its Therapeutic Applications. Curr. Neuropharmacol..

[16] Ambriz-Tututi M., Rocha-González H.I., Castañeda-Corral G., Araiza-Saldaña C.I., Caram-Salas N.L., Cruz S.L., Granados-Soto V. (2009). Role of Opioid Receptors in the Reduction of Formalin-Induced Secondary Allodynia and Hyperalgesia in Rats. Eur. J. Pharmacol..

[17] Quinõnez-Bastidas G.N., Pineda-Farias J.B., Flores-Murrieta F.J., Rodriguez-Silverio J., Reyes-Garcia J.G., Godínez-Chaparro B., Granados-Soto V., Rocha-Gonzalez H.I. (2018). Antinociceptive Effect of (−)-Epicatechin in Inflammatory and Neuropathic Pain in Rats. Behav. Pharmacol..

[18] Harton L.R., Richardson J.R., Armendariz A., Nazarin A. (2017). Dissociation of Morphine Analgesic Effects in the Sensory and Affective. Brain Res..

[19] Zimmermann M. (1983). Guest Editorial Ethical Guidelines for Investigations of Experimental Pain in Conscious Animals. Pain.

[20] (2001). Especificaciones Técnicas Para La Producción, Cuidado y Uso de Los Animales de Laboratorio.

[21] du Sert N.P., Hurst V., Ahluwalia A., Alam S., Avey M.T., Baker M., Browne W.J., Clark A., Cuthill I.C., Dirnagl U. (2020). The Arrive Guidelines 2.0: Updated Guidelines for Reporting Animal Research. PLoS Biol..

[22] Hickman D.L. (2021). Wellbeing of Mice Euthanized with Carbon Dioxide in Their Home Cage as Compared with an Induction Chamber. J. Am. Assoc. Lab. Anim. Sci..

[23] Dubuisson D., Dennis S.G. (1977). The Formalin Test: A Quantitative Study of the Analgesic Effects of Morphine, Meperidine, and Brain Stem Stimulation in Rats and Cats. Pain.

[24] Wheeler-Aceto H., Porreca F., Cowan A. (1990). The Rat Paw Formalin Test: Comparison of Noxious Agents. Pain.

[25] Cervantes-Durán C., Sánchez-Serrano E., Gauthereau-Torres M.Y., Ortega-Varela L.F. (2021). Evaluation of Antinociceptive Effect of the Ketorolac-Topiramate Combination in the Rat Formalin Test. Bull. Pharm. Sci. Assiut.

[26] Kuthati Y., Lin S.H., Chen I.J., Wong C.S. (2019). Melatonin and Their Analogs as a Potential Use in the Management of Neuropathic Pain. J. Formos. Med. Assoc..

[27] Arreola-Espino R., Urquiza-Marín H., Ambriz-Tututi M., Araiza-Saldaña C.I., Caram-Salas N.L., Rocha-González H.I., Mixcoatl-Zecuatl T., Granados-Soto V. (2007). Melatonin Reduces Formalin-Induced Nociception and Tactile Allodynia in Diabetic Rats. Eur. J. Pharmacol..

[28] Suarez-Mendez S., Tovilla-Zarate C.A., Ortega-Varela L.F., Bermudez-Ocaña D.Y., Blé-Castillo J.L., González-Castro T.B., Zetina-Esquivel A.M., Diaz-Zagoya J.C., Esther Juárez-Rojop I. (2017). Isobolographic Analyses of Proglumide–Celecoxib Interaction in Rats with Painful Diabetic Neuropathy. Drug Dev. Res..

[29] Tallarida R. (2001). Drug Synergism: Its Detection and Applications. J. Pharmacol. Exp. Ther..

[30] Tallarida R.J. (2000). Drug Synergism and Dose-Effect Data Analysis.

[31] Ortega-Varela L.F., Herrera J.E., Caram-Salas N.L., Rocha-González H.I., Granados-Soto V. (2007). Isobolographic Analyses of the Gabapentin-Metamizol Combination after Local Peripheral, Intrathecal and Oral Administration in the Rat. Pharmacology.

[32] Zúñiga-Romero A., Ponce-Chávez M.K., Gauthereau-Torres M.Y., Ortega-Varela L.F. (2014). Combination of Diacerhein and Antiepileptic Drugs after Local Peripheral, and Oral Administration in the Rat Formalin Test. Drug Dev. Res..

[33] Stanley J.L., Lincoln R.J., Brown T.A., McDonald L.M., Dawson G.R., Reynolds D.S. (2005). The Mouse Beam Walking Assay Offers Improved Sensitivity over the Mouse Rotarod in Determining Motor Coordination Deficits Induced by Benzodiazepines. J. Psychopharmacol..

[34] Tjølsen A., Berge O.G., Hunskaar S., Rosland J.H., Hole K. (1992). The Formalin Test: An Evaluation of the Method. Pain.

[35] Abbott F.V., Franklin K.B., Westbrook R.F. (1995). The Formalin Test: Scoring Properties of the First and Second Phases of the Pain Response in Rats. Pain.

[36] Rena G., Pearson E.R., Sakamoto K. (2013). Molecular Mechanism of Action of Metformin: Old or New Insights?. Diabetologia.

[37] Rena G., Hardie D.G., Pearson E.R. (2017). The Mechanisms of Action of Metformin. Diabetologia.

[38] Foretz M., Guigas B., Viollet B. (2019). Understanding the Glucoregulatory Mechanisms of Metformin in Type 2 Diabetes Mellitus. Nat. Rev. Endocrinol..

[39] Pernicova I., Korbonits M. (2014). Metformin-Mode of Action and Clinical Implications for Diabetes and Cancer. Nat. Rev. Endocrinol..

[40] Cao X.J., Wu R., Qian H.Y., Chen X., Zhu H.Y., Xu G.Y., Sun Y.Z., Zhang P.A. (2021). Metformin Attenuates Diabetic Neuropathic Pain via AMPK/NF-ΚB Signaling Pathway in Dorsal Root Ganglion of Diabetic Rats. Brain Res..

[41] Hyun B., Shin S., Lee A., Lee S., Song Y., Ha N.-J., Cho K.-H., Kim K. (2013). Metformin Down-Regulates TNF-α Secretion via Suppression of Scavenger Receptors in Macrophages. Immune Netw..

[42] Wei J., Wei Y., Huang M., Wang P., Jia S. (2022). Is Metformin a Possible Treatment for Diabetic Neuropathy?. J. Diabetes.

[43] Pecikoza U., Lasica A., Nastić K., Dinić M., Jasnić N., Micov A., Đorđević J., Stepanović-Petrović R., Tomić M. (2025). Metformin Reduces Inflammatory Nociception in Mice through a Serotonin-Dependent Mechanism. Eur. J. Pharmacol..

[44] Duan D., Wu X., Ali U., Wang D., Li X., Liu R., Ma L., Mao Y., Ma Y. (2024). Metformin Alleviates Pain States by Regulating the Balance of Spinal Synaptic Transmission. J. Integr. Neurosci..

[45] Song Y., Wu Z., Zhao P. (2022). The Effects of Metformin in the Treatment of Osteoarthritis: Current Perspectives. Front. Pharmacol..

[46] Taylor A., Westveld A.H., Szkudlinska M., Guruguri P., Annabi E., Patwardhan A., Price T.J., Yassine H.N. (2013). The Use of Metformin Is Associated with Decreased Lumbar Radiculopathy Pain. J. Pain Res..

[47] Smith B., Ang D. (2015). Metformin: Potential Analgesic?. Pain Med..

[48] Guzmán-Priego C.G., Méndez-Mena R., Baños-González M.A., Araiza-Saldaña C.I., Castañeda-Corral G., Torres-López J.E. (2017). Antihyperalgesic Effects of Indomethacin, Ketorolac, and Metamizole in Rats: Effects of Metformin. Drug Dev. Res..

[49] Pecikoza U.B., Tomić M.A., Micov A.M., Stepanović-Petrović R.M. (2017). Metformin Synergizes with Conventional and Adjuvant Analgesic Drugs to Reduce Inflammatory Hyperalgesia in Rats. Anesth. Analg..

[50] Melemedjian O.K., Asiedu M.N., Tillu D.V., Sanoja R., Yan J., Lark A., Khoutorsky A., Johnson J., Peebles K.A., Lepow T. (2011). Targeting Adenosine Monophosphate-Activated Protein Kinase (AMPK) in Preclinical Models Reveals a Potential Mechanism for the Treatment of Neuropathic Pain. Mol. Pain.

[51] Price T.J., Dussor G. (2013). AMPK: An Emerging Target for Modification of Injury-Induced Pain Plasticity. Neurosci. Lett..

[52] Fatemi I., Amirteimoury M., Shamsizadeh A., Kaeidi A. (2018). The Effect of Metformin on Morphine Analgesic Tolerance and Dependence in Rats. Res. Pharm. Sci..

[53] Inyang K.E., Szabo-Pardi T., Wentworth E., McDougal T.A., Dussor G., Burton M.D., Price T.J. (2019). The Antidiabetic Drug Metformin Prevents and Reverses Neuropathic Pain and Spinal Cord Microglial Activation in Male but Not Female Mice. Pharmacol. Res..

[54] Ortiz M.I. (2013). Synergistic Interaction between Metformin and Sulfonylureas on Diclofenac-Induced Antinociception Measured Using the Formalin Test in Rats. Pain Res. Manag..

[55] Emet M., Ozcan H., Ozel L., Yayla M., Halici Z., Hacimuftuoglu A. (2016). Bir Melatonin Derlemesi, Reseptörleri ve Ilaçları. Eurasian J. Med..

[56] Scarabelot V.L., Medeiros L.F., de Oliveira C., Adachi L.N.S., de Macedo I.C., Cioato S.G., de Freitas J.S., de Souza A., Quevedo A., Caumo W. (2016). Melatonin Alters the Mechanical and Thermal Hyperalgesia Induced by Orofacial Pain Model in Rats. Inflammation.

[57] Xie S., Fan W., He H., Huang F. (2020). Role of Melatonin in the Regulation of Pain. J. Pain Res..

[58] Chaudhry S.R., Stadlbauer A., Buchfelder M., Kinfe T.M. (2021). Melatonin Moderates the Triangle of Chronic Pain, Sleep Architecture and Immunometabolic Traffic. Biomedicines.

[59] Mantovani M., Kaster M.P., Pertile R., Calixto J.B., Rodrigues A.L.S., Santos A.R.S. (2006). Mechanisms Involved in the Antinociception Caused by Melatonin in Mice. J. Pineal Res..

[60] Murad H., Ayuob N. (2015). Co-Administration of Pioglitazone Improves Fluoxetine’s Antinociceptive, Neuroprotective, and Antidepressant Effects in Chronic Constriction Injury in Rats. Pain Physician.

[61] Dasuni Wasana P.W., Hasriadi, Muangnoi C., Vajragupta O., Rojsitthisak P., Rojsitthisak P., Towiwat P. (2022). Curcumin and Metformin Synergistically Modulate Peripheral and Central Immune Mechanisms of Pain. Sci. Rep..

[62] El-Haddad M.E., El-Refaie W.M., Hammad G.O., EL-Massik M.A. (2024). Targeted Non-Invasive Metformin-Curcumin Co-Loaded Nanohyaluosomes Halt Osteoarthritis Progression and Improve Articular Cartilage Structure: A Preclinical Study. Int. J. Pharm..

[63] Qin H.M., Luo Z.K., Zhou H.L., Zhu J., Xiao X.Y., Xiao Y., Zhuang T., Zhang Z.G. (2024). No TitleNovel Drug-Drug Salt Crystals of Metformin with Ibuprofen or Naproxen: Improved Solubility, Dissolution Rate, and Synergistic Antinociceptive Effects. Int. J. Pharm..

[64] Pang C.S., Tsang S.F., Yang J.C. (2001). Effects of Melatonin, Morphine and Diazepam on Formalin-Induced Nociception in Mice. Life Sci..

[65] Hemati K., Pourhanifeh M.H., Dehdashtian E., Fatemi I., Mehrzadi S., Reiter R.J., Hosseinzadeh A. (2021). Melatonin and Morphine: Potential Beneficial Effects of Co-Use. Fundam. Clin. Pharmacol..

[66] Alavez-Pérez N., Patiño-Camacho I.S., Granados-Soto V., Déciga-Campos M. (2022). Melatonin Synergizes with the Antinociceptive Effect of N-Palmitoylethanolamide and Paracetamol. Pharmazie.

[67] Terribili R., Vallifuoco G., Bardelli M., Frediani B., Gentileschi S. (2024). A Fixed Combination of Palmitoylethanolamide and Melatonin (PEATONIDE) for the Management of Pain, Sleep, and Disability in Patients with Fibromyalgia: A Pilot Study. Nutrients.

[68] Gilron I., Debow C., Elkerdawy H., Khan J.S., Salomons T.V., Duggan S., Tu D., Holden R.R., Milev R., Buckley D.N. (2024). PRECISE Trial (Pain RElief Combination Intervention StratEgies): Protocol for the Clinical Trial of a Pregabalin-Melatonin Combination for Fibromyalgia. BMJ Open.

[69] Çakırgöz E., Durdağı G., Öz Oyar E. (2025). Enhanced Analgesia: Synergistic Effects of Melatonin and Tramadol on Acute Thermal Nociception in Wistar Rats via Tail-Flick and Hot-Plate Tests. Behav. Brain Res..

[70] Hussain S.A., Khadim H.M., Khalaf B.H., Ismail S.H., Hussein K.I., Sahib A.S. (2006). Effects of Melatonin and Zinc on Glycemic Control in Type 2 Diabetic Patients Poorly Controlled with Metformin. Saudi Med. J..

[71] Dantas-Ferreira R.F., Raingard H., Dumont S., Schuster-Klein C., Guardiola-Lemaitre B., Pevet P., Challet E. (2018). Melatonin Potentiates the Effects of Metformin on Glucose Metabolism and Food Intake in High-fat-fed Rats. Endocrinol. Diabetes Metab..

[72] Lemos A.J.J.M., Peixoto C.A., Teixeira Á.A.C., Luna R.L.A., Rocha S.W.S., Santos H.M.P., Silva A.K.S., Nunes A.K.S., Wanderley-Teixeira V. (2014). Effect of the Combination of Metformin Hydrochloride and Melatonin on Oxidative Stress before and during Pregnancy, and Biochemical and Histopathological Analysis of the Livers of Rats after Treatment for Polycystic Ovary Syndrome. Toxicol. Appl. Pharmacol..

[73] Lombardi L.A., Mattos L.S., Espindula A.P., Simões R.S., Sasso G.R.D.S., Simões M.J., Soares-Jr J.M., Florencio-Silva R. (2024). Effects of Melatonin and Metformin on the Ovaries of Rats with Polycystic Ovary Syndrome. F S Sci..

[74] Man’Cheva T.A., Demidov D.V., Plotnikova N.A., Kharitonova T.V., Pashkevich I.V., Anisimov V.N. (2011). Melatonin and Metformin Inhibit Skin Carcinogenesis and Lipid Peroxidation Induced by Benz(a)Pyrene in Female Mice. Bull. Exp. Biol. Med..

[75] Deriabina O.N., Plotnikova N.A., Anisimov V.N. (2010). Melatonin and Metformin Inhibit Skin Carcinogenesis Induced by Benz(a)Pyrene in Mice. Vopr. Onkol..

[76] Brown R.E., Buryanek J., McGuire M.F. (2017). Metformin and Melatonin in Adrenocortical Carcinoma: Morphoproteomics and Biomedical Analytics Provide Proof of Concept in a Case Study. Ann. Clin. Lab. Sci..

[77] Kurhaluk N., Bojková B., Winklewski P.J. (2018). Liver Antioxidant and Aerobic Status Improves after Metformin and Melatonin Administration in a Rat Model of High-Fat Diet and Mammary Carcinogenesis. Can. J. Physiol. Pharmacol..

[78] Bojková B., Kajo K., Kubatka P., Solár P., Péč M., Adamkov M. (2019). Metformin and Melatonin Improve Histopathological Outcome of NMU-Induced Mammary Tumors in Rats. Pathol. Res. Pract..

[79] Najafi M., Cheki M., Hassanzadeh G., Amini P., Shabeeb D., Musa A.E. (2019). The Radioprotective Effect of Combination of Melatonin and Metformin on Rat Duodenum Damage Induced by Ionizing Radiation: A Histological Study. Adv. Biomed. Res..

[80] Tajabadi E., Javadi A., Azar N.A., Najafi M., Shirazi A., Shabeeb D., Musa A.E. (2020). Radioprotective Effect of a Combination of Melatonin and Metformin on Mice Spermatogenesis: A Histological Study. Int. J. Reprod. Biomed..

[81] Nair A.B., Jacob S. (2016). A Simple Practice Guide for Dose Conversion between Animals and Human. J. Basic Clin. Pharm..

[82] Blanchard O.L., Smoliga J.M. (2015). Translating Dosages from Animal Models to Human Clinical Trials--Revisiting Body Surface Area Scaling. FASEB J..

[83] Ray M., Mediratta P.K., Mahajan P., Sharma K.K. (2004). Evaluation of the Role of Melatonin in Formalin-Induced Pain Response in Mice. Indian J. Med. Sci..

[84] Lopez-Canul M., Palazzo E., Dominguez-Lopez S., Luongo L., Lacoste B., Comai S., Angeloni D., Fraschini F., Boccella S., Spadoni G. (2015). Selective Melatonin MT2 Receptor Ligands Relieve Neuropathic Pain through Modulation of Brainstem Descending Antinociceptive Pathways. Pain.

[85] Cheng J.T., Huang C.C., Liu I.M., Tzeng T.F., Chih J.C. (2006). Novel Mechanism for Plasma Glucose-Lowering Action of Metformin in Streptozotocin-Induced Diabetic Rats. Diabetes.

[86] Shavali S., Ho B., Govitrapong P., Sawlom S., Ajjimaporn A., Klongpanichapak S., Ebadi M. (2005). Melatonin Exerts Its Analgesic Actions Not by Binding to Opioid Receptor Subtypes but by Increasing the Release of β-Endorphin an Endogenous Opioid. Brain Res. Bull..

[87] Russe O.Q., Möser C.V., Kynast K.L., King T.S., Stephan H., Geisslinger G., Niederberger E. (2013). Activation of the AMP-Activated Protein Kinase Reduces Inflammatory Nociception. J. Pain.

[88] Price T.J., Das V., Dussor G. (2016). Adenosine Monophosphate-Activated Protein Kinase (AMPK) Activators For the Prevention, Treatment and Potential Reversal of Pathological Pain. Curr. Drug Targets.

[89] Motilva V., García-Mauriño S., Talero E., Illanes M. (2011). New Paradigms in Chronic Intestinal Inflammation and Colon Cancer: Role of Melatonin. J. Pineal Res..

[90] McCarty M.F., Assanga S.B.I., Luján L.L., O’keefe J.H., Dinicolantonio J.J. (2021). Nutraceutical Strategies for Suppressing Nlrp3 Inflammasome Activation: Pertinence to the Management of COVID-19 and Beyond. Nutrients.

[91] Liu Q., Su L.Y., Sun C., Jiao L., Miao Y., Xu M., Luo R., Zuo X., Zhou R., Zheng P. (2020). Melatonin Alleviates Morphine Analgesic Tolerance in Mice by Decreasing NLRP3 Inflammasome Activation. Redox Biol..

[92] Ramirez A.D., Gotter A.L., Fox S.V., Tannenbaum P.L., Yao L., Tye S.J., McDonald T., Brunner J., Garson S.L., Reiss D.R. (2013). Dual Orexin Receptor Antagonists Show Distinct Effects on Locomotor Performance, Ethanol Interaction and Sleep Architecture Relative to Gamma-Aminobutyric Acid-A Receptor Modulators. Front. Neurosci..

